# Iodine nutrition among pregnant women in the Faroe Islands

**DOI:** 10.1017/S0007114524001697

**Published:** 2024-08-28

**Authors:** Herborg Líggjasardóttir Johannesen, Stig Andersen, Stine Linding Andersen, Solrunn Hansen, Jóhanna Petursdóttir, Pál Weihe, Marin Strøm, Maria Skaalum Petersen, Anna Sofía Veyhe

**Affiliations:** 1 Department of Endocrinology and Medicine, The National Hospital of the Faroe Islands, Torshavn, Faroe Islands; 2 Centre of Health Science, Faculty of Health Science, the University of the Faroe Islands, Torshavn, Faroe Islands; 3 Steno Diabetes Centre Faroe Islands, The National Hospital of the Faroe Islands, Torshavn, Faroe Islands; 4 Department of Clinical Medicine, Aalborg University, Aalborg, Denmark; 5 Arctic Health Research Centre, Aalborg University Hospital, Aalborg, Denmark; 6 Greenland Centre for Health Research, University of Greenland, Nuuk, Greenland; 7 Department of Clinical Biochemistry, Aalborg University Hospital, Aalborg, Denmark; 8 Department of Health and Care Sciences, UiT The Arctic University of Norway, Tromso, Norway; 9 Department of Research, National Hospital of the Faroe Islands, Torshavn, Faroe Islands

**Keywords:** Iodine status, Urinary iodine excretion, Health survey, Faroe Islands, Artic society

## Abstract

Ensuring adequate iodine nutrition during pregnancy is crucial for fetal brain development. Thus, the WHO recommend monitoring iodine nutrition in pregnant women. With changing dietary habits and declining iodine intake in coastal populations, iodine nutrition in pregnant Faroese women was a focus in newly established pregnancy cohorts. This study aimed to monitor the iodine status of pregnant women in the Faroe Islands by assessing urinary iodine concentration (UIC) and maternal iodine intake. For 2 years, all pregnant women were invited to participate in a nationwide study. Participants completed questionnaires addressing personal and lifestyle factors, supplement intake and dietary habits, Additionally, they provided spot urine samples for UIC measurements. Iodine was measured spectrophotometrically using the ceri/arsen method after alkaline-ashing. Among the 1030 invited, 654 participated and 647 provided a spot-urine sample. The average age was 30·4 years (18–47 years). The overall median UIC was 110 µg/l, declined from 117 to 101 µg/l over 2 years (*P* = 0·004). UIC was significantly impacted by diet. Women consuming fish and eggs had a higher median UIC compared with those whose primary iodine source was dairy: fish-dinner, 151 µg/l; dairy products, 112 µg/l (*P* < 0·001). Furthermore, there was a positive association between maternal age, reported intake of iodine-containing supplements and the UIC. This nationwide study of pregnant Faroese women found UIC below the WHO-recommended cut-off for pregnant women and decreasing with time. This decline highlights the importance of continuous monitoring to prompty identify shifts in iodine status, enabling timely intervention to address emerging deficiencies.

## Graphical abstract

**Figure:**
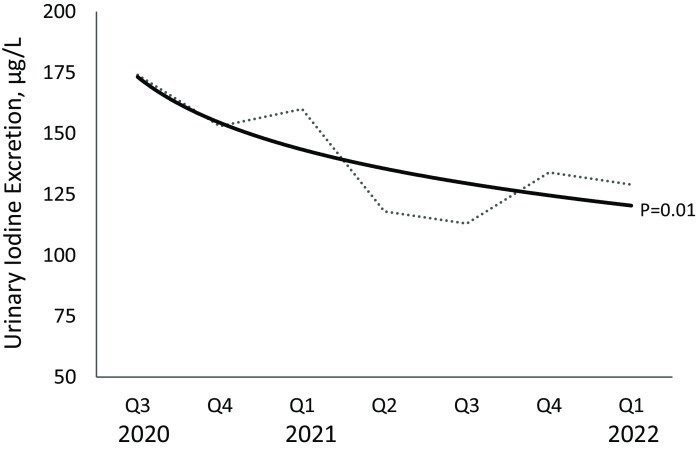

Iodine deficiency is a global public health issue^(1)^ that may lead to various disorders, depending on its severity and population characteristics. Iodine nutrition is critical during fetal and neonatal periods^(2,3)^. Iodine is necessary for synthesising thyroid hormones during pregnancy and for growth and neurodevelopment^(4)^. Hence, iodine is an essential element acquired from the diet and is crucial for both the mother and child^(5)^.

The dietary demand for iodine increases during pregnancy due to the elevated maternal renal clearance of iodine and thyroid hormone production, fetoplacental transport and the initiation of breast milk production^(5)^.

Urinary iodine concentration (UIC) is an indirect marker of iodine intake, which is recommended for settling population iodine intake as 90 % of the daily intake of iodine is excreted in the urine^(1)^. Other biomarkers, such as serum thyroglobulin or thyroid hormone levels,^(6)^ offer complementary information but are influenced by factors beyond iodine intake such as thyroid diseases and medication use. Pre-pregnancy iodine status is essential for preventing gestational iodine deficiency, and the WHO classifies iodine intake in a group of pregnant women as insufficient when the median UIC is below 150 µg/l and adequate if 150–250 µg/l, corresponding to an intake of 250 µg/d^(1,7)^. The Nordic Nutrition Recommendation (NNR) focuses on adequate intake set as 200 µg/d for pregnant women in line with recommendations from the European Food Safety Authority^(8,9)^. Adequate iodine consumption should rely on diet rather than dietary supplements^(10)^. However, it has been suggested that pregnant women take iodine-containing supplements if the median population UIC is below 100 µg/l^(10)^. Nonetheless, the need for iodine supplementation during pregnancy in moderately or mildly iodine-deficient areas remains debatable^(10,11)^.

Salt iodisation accounts for 88 % of households worldwide^(12)^ and remains the most efficient method for preventing iodine deficiency disorder. However, iodine deficiency remains a major public health concern^(13–16)^.

The Danish iodine fortification program contributes to iodine intake in the Faroe Islands because imported salt originates mostly from Denmark^(17)^. A nationwide study among Faroese teenagers demonstrated that this group was iodine-replete by the turn of the millennium^(18)^. A decade later, a decrease in iodine intake was suggested by a median UIC of 101 µg/l in Faroese adults,^(19)^ with the lowest UIC in Faroese women. In addition, a low median UIC among pregnant women in Denmark^(20)^ raises a concern regarding iodine deficiency during pregnancy in the Faroe Islands. Thus, the present study aimed to assess the iodine status of pregnant women in the Faroe Islands, an area known for its unique dietary and environmental factors that may impact iodine intake. Additionally, we sought to elucidate the dietary components that influence iodine intake within this population by assessing both iodine excretion and dietary patterns^(17)^ to inform public health interventions and dietary recommendations tailored to the needs of pregnant women in the Faroe Islands and similar coastal populations on the North Atlantic.

## Materials and methods

### Area of investigation

The Faroe Islands are an island community in the North Atlantic Ocean (61°53′52″ N, 6°55′43″ W), 200 miles (360 km) northwest of Scotland and 370 miles (590 km) west of Norway. They are part of the Nordic countries with similarities in language, welfare system and lifestyle, but with some dietary peculiarities^(18)^, such as seabirds, pilot whales and other seafood with a high iodine content that could support sufficient dietary iodine intake^(17)^.

On the Faroe Islands, iodised and non-iodised salts are available in most grocery stores. Local bakeries mostly use iodine-fortified salt for bread production^(17,19)^, but voluntarily. While the Faroese health authorities follow Danish recommendations regarding adequate dietary intake, including those for pregnant women, there are no official recommendations regarding the sources of iodine intake.

### Participant recruitment

All pregnant women in the Faroe Islands were invited to participate aiming to comprehensively assess the iodine status of the entire pregnant population. We aimed for a sample size with a minimum of 489 spot urine samples to settle the median UIC with 5 % precision^(21)^. Pregnant women were recruited between June 2020 and April 2022, aiming at women around gestational week-18 as part of the recent birth eCohort-6 within the Department of Research at the National Hospital of the Faroe Islands. Eligible participants were asked for permission by the midwives during their first midwife appointment to be contacted by the study coordinator. A total of 1030 pregnant women were invited to participate in the study. Of these, 246 declined participation, 71 accepted and subsequently withdrew and 61 were nonresponsive, leading to a participation rate of 63 %, including 654 pregnant women.

### Questionnaires

At the study entry, participants answered questionnaires on dietary habits, personal information and lifestyle. The questionnaires were completed online.

The FFQ used to assess the dietary intake in the present study aimed at mapping iodine sources for the past 7 days. The dietary questions were developed from dietary inquiries utilised in a validated FFQ capturing the previous 12 months’ intake^(22)^ as informed by our report on the iodine content of Faroese food items^(17)^. Dietary questions included milk, cocoa milk, yoghurt and other sour milk products; milk for coffee or tea; cheese and spread cheese; lean and fatty fish and fish products for dinners and fish products such as cold-cuts, roe, shellfish, eggs and bread (added to the questionnaire from May 2021). Groupings were categorised based on iodine concentrations sourced from the Norwegian Food Composition Table^(23)^. Women were asked to report their intake yesterday and today (yes/no), as well as during the previous 7 days, with six frequency options ranging from 0 to 4+ times per day. As for the Faroese dietary peculiarities, women were asked to report their intake of whale meat, whale blubber sea birds and seaweed over the past 12 months, and since the beginning of pregnancy. The use of fish oil and iodine-containing supplements in the previous week, including the name of the product and the date of the most recent intake.

In addition, the questionnaire covered marital status, schooling, education, health status, medication use, smoking, snuffing and alcohol intake during the past week and today. Nineteen women who reported a diagnosis of thyroid disease and an additional three taking thyroxine were included in the analyses. Finally, participants reported their height, weight and age. BMI was calculated as the weight in kilograms divided by the height in metres squared.

### Urine sampling and analysis

Among 654 participants, 647 provided a first-morning void sample, collected at home, at study entry. All samples were stored in iodine-free polyethylene containers at –80°C at the Department of Research, the National Hospital of the Faroe Islands, Torshavn, the Faroe Islands, until analysis.

Iodine was measured at the Iodine Laboratory at Aalborg University Hospital using the Sandell-Kolthoff reaction, modified according to Wilson and van Zyl^(24)^ (EQUIP quality control, E-117). The principle is the evaporation and alkaline ashing of the sample, followed by resuspension and measurement of iodine by spectrophotometric detection of the catalytic role of iodine in the reduction of ceric ammonium sulphate in the presence of arsenious acid^(25)^. We aimed for a sample size of a minimum of 489 spot urine samples to settle the median UIC with 5 % precision^(24)^.

### Statistical analysis

Statistical analyses were performed using SPSS Statistics for Windows (version 28·0; SPSS Inc.).

The frequency of intake was quantified from the dietary questions of the previous week’s intake, and the intake during the present and previous days was kept as binary yes/no answers. Continuous variables were analysed for normal distribution by visually inspecting QQ plots and using the Kolmogorov–Smirnov test. Means, standard deviations, medians and interquartile ranges were provided for continuous variables. The number of cases and percentages (%) in each category are reported for categorical variables. Two groups were compared using Student’s *t* test or the non-parametric Mann–Whitney test and ANOVA and/or the post hoc Bonferroni test or Kruskal–Wallis test was applied when comparing several groups. The *χ* ^*2*^-test was used for categorical variables. Univariable linear regression was applied to further explore the impact on UIC [log-transformed; base 10 logarithm (log_10_ *x*)] with the following background explanatory variables: age (both continuous and categorical), pre-pregnancy BMI (both continuous and categorical), educational attainment (up to secondary school or over the secondary school), born in the Faroe Islands (yes/no) and living in the capital area (yes/no). The dietary explanatory variables were entered into the categories ‘yes/no’ for yesterday/today’s intake and the frequency of intake within the past week and included the following five categories: fish products, dairy products, cheese, eggs and bread. Other nutritive items recorded as ‘yes/no’ included iodised salt for cooking and supplements containing iodine. Subsequently, predictors that were statistically significant in the univariable analyses were entered into the multivariable linear regression models with forward inclusion. The model check revealed normally distributed residuals, homoscedasticity and no multicollinearity.

To further explore dietary intake, we drew on a dimension reduction using principal component analysis based on Eigenvalues > 1 and Varimax rotation to produce a smaller number of uncorrelated variables while retaining most of the variance of the raw data. The Kaiser–Meyer–Olkin test was used to evaluate the data for factor analysis. Analyses were performed using a combination of the five dietary variables for the previous week’s and yesterday’s/today’s intake and intake of whale meat, whale blubber and seabirds during pregnancy. The combination of dietary components explaining the highest variation and Kaiser–Meyer–Olkin test value was the previous week’s intake of milk products, cheese, fish dinners, fish cold cuts and eggs. The new axes were explored using linear regression models explaining the UIC (log-transformed).

The sampling period was grouped into four 6-month intervals, and differences with time were tested using the Kruskal–Wallis test comparing groups of six months: June–November 2020; December 2020–May 2021; June–November 2021 and December 2021–April 2022. This was elaborated for the analysis of time trends in UIC in a linear regression model with adjustments for age, gestational week, BMI, iodine intake, intake of fish and dairy products and total food intake. The sampling periods were also included as dummy variables and the fractional change in UIC (log-transformed) was compared with the first period (June 2020–November 2020). Further, a F-test was used to compare the log-UIC levels in the four periods. Finally, the intake of all food recorded for fish and dairy products was included in the analysis.

## Results

### Participant characteristics and dietary intakes

Participant characteristics are listed in Table 1 and detailed in online Supplementary Table S1. Nearly half of the study population lived in the capital area, with 86 % born on the Faroe Islands. The pre-pregnancy median BMI was 26·4 kg/m^2^ with two out of three being overweight or obese. Iodised salt was used by 56 % of the women, and their dietary habits are detailed in online Supplementary Table S2.

**Table 1.** Participant characteristics and urinary iodine concentration (UIC, µg/l) according to these characteristics and dietary intake early in the second trimester in Faroese pregnant women (Median values and interquartile ranges; 95 % confidence intervals)

Variables	Groups	*n*	UIC Median	IQR	95 % CI*	*P* §
Overall		647	110	76–176	102, 116	0·04
Age group, years	<25	76	96	73–161	85, 115	
	25–29	195	101	69–156	90, 107	
	30–34	215	116	79–181	101, 128	
	35–39	112	116	77–184	102, 137	
	≥40	19	152	86–205	100, 204	
Education	Up to upcondary	199	97	73–156	90, 111	0·05
	Skilled and higher	435	113	76–182	105, 124	
Smoke or snuff†	Yes	56	106	58–179	90, 140	0·5
	No	564	108	76–172	101, 115	
Alcohol intake†	Yes	15	95	46–148	46, 144	0·4
	No	574	109	75–175	102, 116	
Country of origin (place of birth)	Faroes	550	110	76–174	103, 119	0·8
	Nordic country	67	103	68–184	85, 125	
	Outside Nordic region	24	95	65–226	68, 199	
Residence	Capital area	290	113	78–180	102, 125	0·1
	Outside capital area (<6000)	349	106	72–176	99, 116	
BMI groups	<24·9‡	218	112	72–181	99, 123	0·9
	25·0–29·9	231	103	76–181	92, 112	
	30·0–34·9	89	115	79–158	101, 129	
	35·0–39·9	42	136	79–163	95, 153	
	≥40·0	14	93	73–160	75, 152	
Vitamin with iodine intake yesterday	Yes	446	115	79–182	106, 125	<0·001
	No	103	92	63–125	82, 105	
Milk products yesterday	Yes	482	112	76–176	105, 120	0·09
	No	141	95	67–169	86, 107	
Cheese yesterday	Yes	311	117	79–182	108, 130	0·002
	No	312	100	70–160	92, 109	
Fish dinner yesterday	Yes	113	151	82–221	120, 177	<0·001
	No	510	103	73–156	97, 110	
Fish cold-cut yesterday	Yes	73	130	83–197	108, 151	0·02
	No	550	105	73–166	100, 113	
Egg yesterday	Yes	136	130	78–196	107, 151	0·02
	No	487	104	73–161	98, 111	
Bread yesterday	Yes	251	99	70–143	90, 110	0·9
	No	30	103	73–143	87, 124	
Seaweed past week	Yes	53	106	76–169	92, 140	0·9
	No	560	107	73–175	100, 113	
Thyroid disease	Yes	19	145	101–192	114, 186	0·05
	No	542	108	75–177	100, 116	

* 95 % CI based on 1000 Bootstrap samples and refers to the median concentration of each group.

† Past week and yesterday/today.

‡ Two subjects had pre-pregnancy BMI < 18·5 and were included in the group with a BMI of 18·5–24·9.

§ *P* values based on the Mann–Whitney U test for two groups and the Kruskal–Wallis test for several groups.

IQR, interquartile range.

### Urinary iodine concentration and its determinants

The overall median UIC was 110 µg/l (IQR 76–176 µg/l), and the distribution of iodine concentration in urine samples is shown in online Supplementary Fig. S1. The UIC was 17 % higher in the older group (< 30 years *v.* ≥ 30 years; *P* = 0·004; online Supplementary Fig. S2), but yesterday’s iodine-containing vitamin intake was similar between the younger (< 30 years) and older (≥ 30 years) participants; 78 % *v.* 81 %, *χ*^2^ = 0·8, *P* = 0·4.

Univariable linear regression analysis showed a positive association between maternal age, higher educational attainment and the outcome of UIC (online Supplementary Table S3). Living outside the capital area was associated with a possible 10 % lower UIC score (*P* = 0·09). Recent intake of cheese, fish and iodine-containing supplements and the previous week’s intake of fish dinners and eggs were all statistically significant predictors of UIC (online Supplementary Table S3). From the multivariable linear regression model, three predictor variables were identified: maternal age (*P* = 0·009), yesterday’s intake of supplements containing iodine (*P* < 0·001) and yesterday’s intake of fish dinner (*P* < 0·001). A significant interaction was detected between age and fish dinners, and by assessing standardised-*β*, maternal age was eliminated from the model because the unit-independent relative contribution to the UIC for fish dinners was 10^*β*^ = 1·29 compared with 10^*β*^ = 1·58 for fish dinners. After eliminating age from the model, the F-values increased from 17·3 to 22·4 (Table 2).

**Table 2.** Multivariable linear regression model assessing the fractional change in UIC (µg/l) per unit change in the independent variable (Ratios and 95 % confidence intervals)

Variable	Group	*n*	Ratio‡	95 % CI	*P*	Standardised-*β*	R^2^	F	*P*
Vitamins*,†		431	1·39	1·22, 1·60	<0·001	1·57	0·08	22·4	<0·001
Fish dinner*		113	1·39	1·21, 1·60	<0·001	1·56			

* Intake yesterday/today (yes).

† Multivitamins containing iodine.

‡ Corresponds to 10^*B*^, with *B* the unstandardised regression coefficient.

UIC, urinary iodine concentration.

### Principal component analyses of dietary habits and the association with urinary iodine concentration

Two new axes appeared from the principal component analysis analysis, including the dietary variables, explaining 51 % of the total variance (online Supplementary Table S4), with Kaiser–Meyer–Olkin = 0·6 and Bartlett’s test *P* < 0·001. The new axes were entered into an univariable linear regression model. Pregnant women with the combined intake of fish, fish products and eggs (PC-1) had an overall higher UIC, see online Supplementary Table S4, and when explored further in the multivariable linear regression model, intake of iodine-containing supplements and higher educational attainment explained 6 % of the total variation, as presented in Table 3. Intake of dairy products (PC-2) was not associated with UIC (*P* = 0·7).

**Table 3.** Multivariable linear regression model assessing the fractional change in UIC (µg/l) per unit change in the independent variable PC-1 (fish, cold-cut, egg) and personal predictors (Ratios and 95 % confidence intervals)

Variable	Group	*n*	Ratio§	95 % CI	*P*	Standardised-*β*	R^2^	F	*P*
PC-1	Fish. cold-cut. eggs*	609	1·07	1·01, 1·14	0·02	1·27	0·06	11·3	<0·001
Vitamins†,‡		431	1·40	1·21, 1·61	<0·001	1·57			
Education	Upper secondary	199	–	–	0·02	–			
	Skilled and higher	435	1·16	1·02, 1·32		1·25			

* Intake during the previous week.

† Intake yesterday/today (yes).

‡ Multivitamins containing iodine.

§ Corresponds to 10^*B*^, with *B* the unstandardised regression coefficient.

UIC, urinary iodine concentration.

### Time-Trend in UIC

**Fig. 1.**
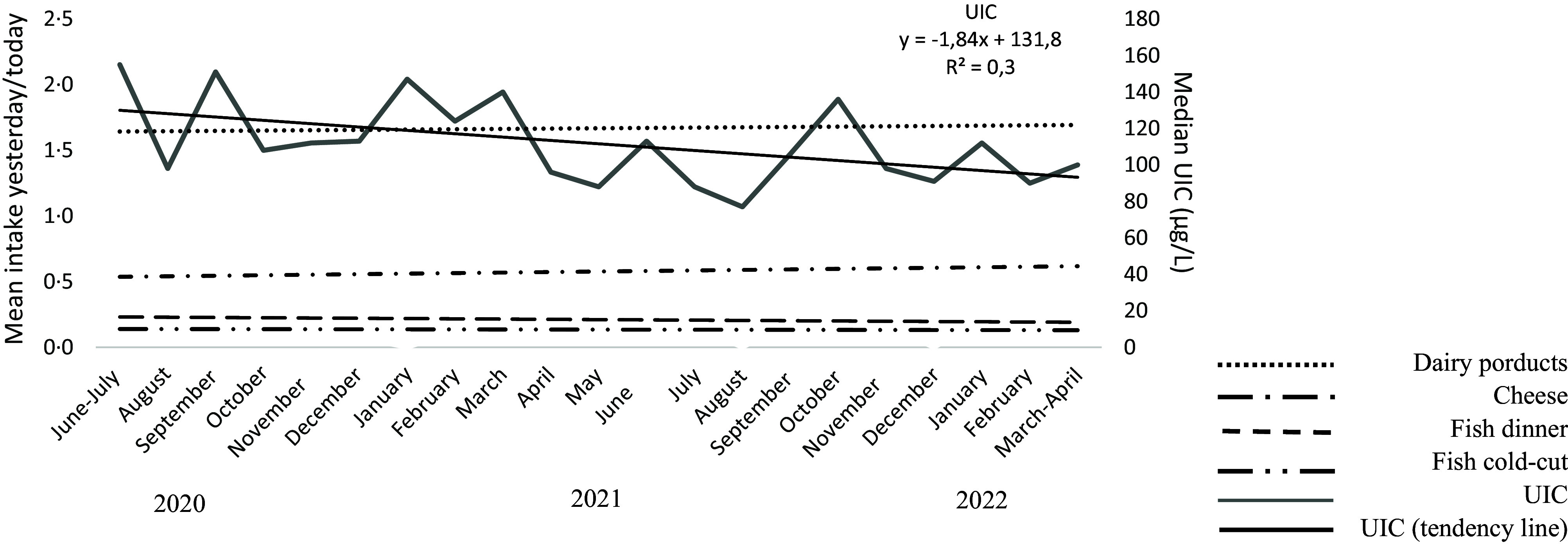
Median urinary iodine concentration (µg/l) by month of urine sampling and dietary intake.

**Fig. 2.**
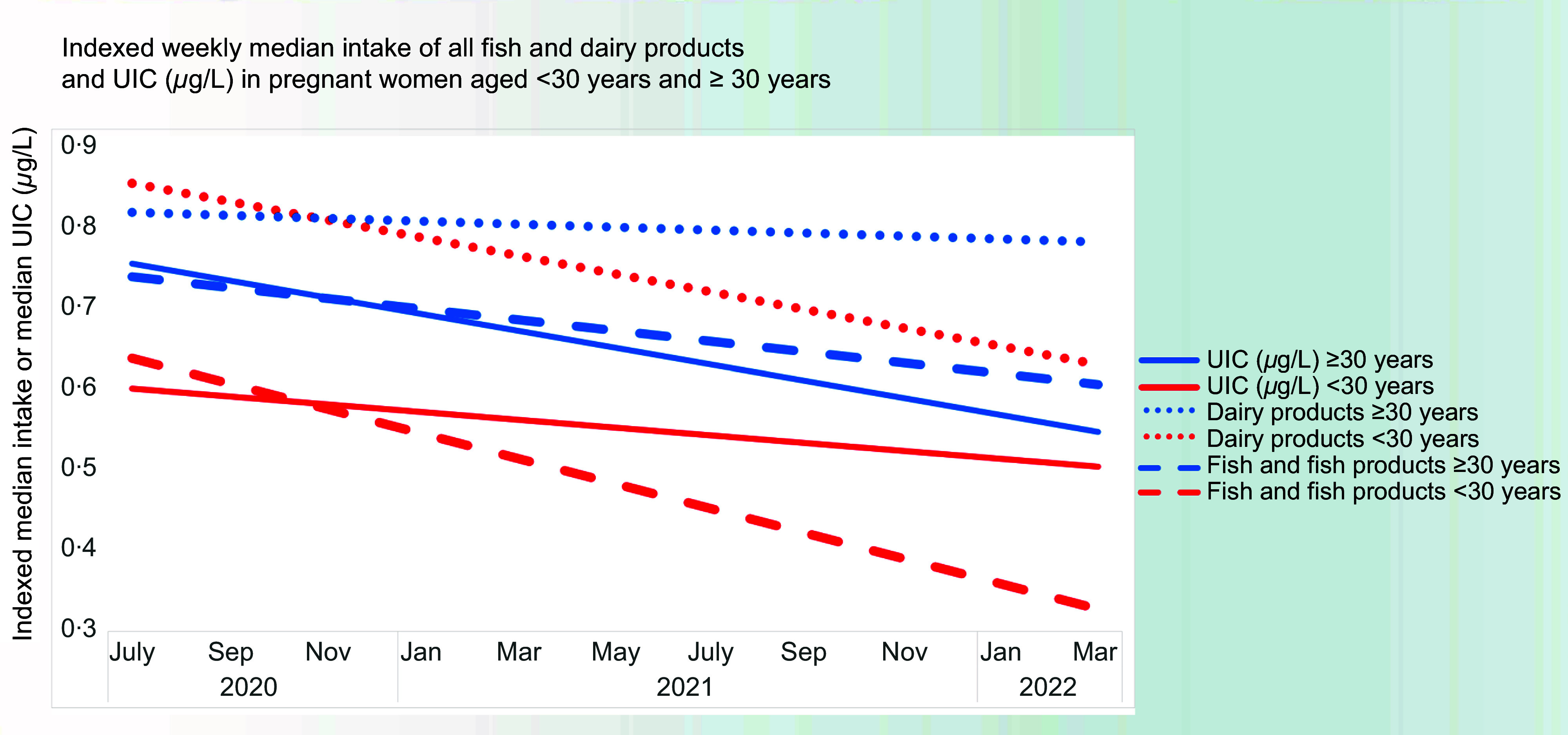
Age-related disparities: lower urinary iodine concentration in younger women (< 30 years) compared with older counterparts (≥ 30 years) (< 30 years *v.* ≥ 30 years, ratio 1·17; *P* = 0·004).

The decrease in UIC over the 2-year sampling period illustrated in the graphical abstract is detailed in Fig. 1. The median UIC was lower late in the sampling period (101 µg/l in samples from December–April 2022) than early in the study (117 µg/l in June–November 2020) (P_M-W_ = 0·004). Figure 2 shows a time-trend in the intake of iodine-containing foods and highlights that the decline in dairy and fish intake is most pronounced in the younger age group. Online Supplementary Table S5 lists the four 6-month sampling intervals with the demographic and dietary characteristics of the participating pregnant women. There were differences with time. When analysed in a linear regression model with the sampling periods included as dummy variables, the fractional change in UIC (log-transformed) compared with the first period (June 2020 to November 2020) was as follows: December 2020 to May 2021, Ratio 10^*B*^ = 0·89, *P* = 0·1; June 2021 to November 2021, ratio 10^*B*^ = 0·79, *P* = 0·002; December 2021 to April 2022, ratio 10^*B*^ = 0·78, *P* = 0·003. The overall model: R^2^ = 0·02, F = 4·4, *P* = 0·005.

## Discussion

Our two main findings were UIC levels below those recommended by the WHO and a decreasing trend over time. The overall median UIC of 110 µg/l among pregnant women in the Faroe Islands was contributed by a lower median UIC of 101 µg/l in those recruited late, compared with a median UIC of 117 µg/l in those recruited in the first year. Our findings classified the overall iodine status among pregnant Faroese women as insufficient according to the WHO, with a trend indicating a decline in UIC over time, and the decrease in consumption of iodine-rich dietary sources seems to pertain mainly to the younger generation.

The UIC among pregnant Faroese women was comparable with that of pregnant women in other areas of the world; China (UIC 130 µg/l)^(26)^ and Nordic countries, including Finland (115 µg/l)^(27)^ and Sweden (101 µg/l)^(28)^, yet slightly above those in Iceland (89 µg/l)^(29)^, Norway (84 µg/l)^(30)^ and Denmark (77 µg/l)^(20)^. Changing dietary patterns with decreasing consumption of dairy and marine products are ongoing in Nordic countries^(31)^. This dietary drift away from iodine-rich foods was associated with a marked decrease in UIC from 180 µg/l to 89 µg/l over a decade among pregnant women in Iceland^(29)^. Similar findings were reported from Norway^(32)^ and Denmark and our results indicate a comparable trend among Faroese pregnant women, which raises the question of the rate of decline in iodine intake.

Urine sampling conducted over 2 years in our study demonstrated the rate of decline in UIC among pregnant Faroese women between 2020 and 2022, with median values of 117 µg/l from June to November 2020 and 101 µg/l from December to April 2022. The rate of decline in UIC among pregnant women varies between Denmark and Iceland. Reports have indicated a decrease in the median UIC of 25 µg/l in Denmark and 100 µg/l in Iceland throughout the decade^(20,29)^. Despite the variation in these rates, they collectively indicate an ongoing decrease, underscoring the importance of undertaking longitudinal studies on iodine nutrition rather than relying solely on cross-sectional studies.

The decrease in median UIC over time may have several explanations including a change in the intake of local foods rich in iodine, as outlined above. The decrease in the median UIC may be linked to factors such as evolving dietary preferences, changing availability during the COVID-19 pandemic, cultural practices, concerns regarding potential contaminants from traditional foods and maternal age. The women above the age of 30 years had more stable dietary patterns with continuously higher fish and dairy intakes throughout the study than the younger women, yet with a similar intake of iodine-containing vitamins and supplements. Conversely, younger women may have different nutritional priorities and lifestyles that affect their iodine intake. Moreover, only half of the study group was aware of the use of iodised salt. The lack of knowledge on the iodine status among pregnant women indicates the need to raise awareness of the importance of iodine nutrition, which should be balanced with information overload in this vulnerable group.

Living conditions in the Faroes resemble those of the coastal populations in the North Atlantic, including Northern Norway and the Orkney and Shetland Islands. We demonstrated a positive association between iodine-rich foods and UIC, particularly the intake of fish and eggs, while our findings concur with the fact that in the Faroe Islands, dairy products have been found to have a low iodine content compared with those in other countries, where the association between dairy intake and UIC is more evident^(17,32,33)^. In addition, Norway and Iceland fortify animal feed with iodine, which increases the iodine content in dairy products^(33,34)^. In contrast, there is no iodine fortification of animal fodder on the Faroe Islands, diminishing the contribution to human iodine intake from dairy products. Our study highlights that the consumption of iodine-rich traditional Faroese foods, such as whale meat, blubber and seabirds, was relatively low, and that the weekly fish intake did not reach the recommendation to support sufficient iodine intake. Thus, the Faroese authorities have two dietary recommendations on pilot whale meat and blubber, one of which lists this as unsuited for human consumption^(35)^, while the other advises pregnant women to avoid whale blubber until after childbirth and to refrain from whale meat consumption three months before conception and during lactation^(36)^. Our findings align with the risk groups for iodine deficiency identified in the Nordic and Baltic countries^(8,31)^: individuals with a low intake of dairy and marine foods in areas without an iodine fortification programme. However, the estimated consumption of dairy and seafood has halved in the general population, including the Faroe Islands, since the turn of the millennium and causes concern, as reported in Iceland, Norway and the UK^(19,29,32,37)^.

In a recent UK cohort study, pregnant women with obesity had a median UIC of 147 µg/l^(38)^. Our study’s notable 63 % prevalence of overweight and obesity prompts careful consideration of its potential impact on the iodine status. However, no direct link between UIC and BMI was identified in our data, perhaps due to a lack of variability in the participants’ BMI, but comparable to Nordic findings^(8)^.

We recently reported on the incidence of thyroid diseases among all Faroese between 2006 and 2018 and found limited iodine deficiency disorders^(39)^. The lack of prevailing iodine deficiency disorders considering the median iodine intake found may give rise to speculation on the WHO delineation of pregnant women as deficient when the median UIC falls below 150 µg/l^(1)^. The NNR recommendation uses a level of adequate intake, which may be easier to comprehend when providing a lower limit of iodine intake to aim for. This extends our reflection on the delineation of adequate iodine intake among pregnant women^(1,40)^. However, further reports are needed to qualify this claim of the approach to prevent iodine deficiency in pregnant women.

The strengths of the present study are attributed to the meticulous planning of the study, a focus on pregnant women in the Faroe Islands, the nationwide design and the recruitment of participants from the coherent Faroese healthcare system. The extended sampling period was used to capture seasonal differences. We saw limited seasonal differences but a distinct time trend. The COVID-19 pandemic coincided with the commencement of our study but was less likely to be a limitation, as we saw a stable participation rate of approximately 65 % throughout the study. Maternal iodine intake was not measured directly as we used UIC as a surrogate marker of iodine intake. This method is commonly employed in epidemiological studies due to practical challenges in the direct measurements. Still, the use of UIC is an indirect marker of iodine intake that reflects recent iodine intake, renal function and hydration status. We collected information on the time lag between food intake and urine collection; however, we were unable to ascertain the precision of these data. Recall bias is possible as dietary habits were collected by self-reported prior intake. Some women may be unaware of the iodine content in certain supplements or may not consider it when reporting their supplement use, which can introduce uncertainty. Finally, the study is of potential relevance to similar coastal populations in the North Atlantic Ocean, even though findings among Faroese women may differ from those among other populations.

### Conclusion

Our nationwide study demonstrated that pregnant Faroese women had insufficient iodine intake according to the WHO classification. The low median UIC observed among pregnant women in the Faroe Islands as well as among pregnant women in other North Atlantic populations without documented cases of iodine deficiency disorders questions the validity of the WHO recommendation for these populations. Finally, the decrease in iodine status over time in our data suggests the need for continuous evaluation of UIC levels in pregnant women to aid health authorities in promptly identifying changes in iodine status, allowing for timely measures to address emerging deficiencies.

## Supporting information

Johannesen et al. supplementary material 1 Johannesen et al. supplementary material

Johannesen et al. supplementary material 2 Johannesen et al. supplementary material

Johannesen et al. supplementary material 3 Johannesen et al. supplementary material

Johannesen et al. supplementary material 4 Johannesen et al. supplementary material

Johannesen et al. supplementary material 5 Johannesen et al. supplementary material

Johannesen et al. supplementary material 6 Johannesen et al. supplementary material

## Supplementary material

For supplementary material/s referred to in this article, please visit https://doi.org/10.1017/S0007114524001697
