# Supplementary material for: Iodine nutrition among pregnant women in the Faroe Islands
Source: Br J Nutr. 2024 Sep 16;132(4):495–502. doi: 10.1017/S0007114524001697 (PMC11499083; doi:10.1017/S0007114524001697)
Supplement: Johannesen et al. supplementary material 1 — Johannesen et al. supplementary material [file S0007114524001697sup001.docx]

**Supplementary figure 1**

Distribution of iodine concentrations in 647 urine samples from Faroese pregnant women at gestational week 20.
