# Supplementary material for: Iodine nutrition among pregnant women in the Faroe Islands
Source: Br J Nutr. 2024 Sep 16;132(4):495–502. doi: 10.1017/S0007114524001697 (PMC11499083; doi:10.1017/S0007114524001697)
Supplement: Johannesen et al. supplementary material 2 — Johannesen et al. supplementary material [file S0007114524001697sup002.docx]

| **Supplementary Table S1.** Personal characteristics of all study participants at enrollment. | | | | | |
| --- | --- | --- | --- | --- | --- |
| **Variables** | **Groups** | **n** | **Mean (SD) or**  ***n (%)*** | **Median** | **Min-Max** |
| Age |  | 625 | 30.4 (5.0) | 30.0 | 18-47 |
| Age groups | <25 |  | *78 (12.5)* |  |  |
|  | 25-29 |  | *197 (31.5)* |  |  |
|  | 30-34 |  | *218 (34.9)* |  |  |
|  | 35-39 |  | *112 (17.9)* |  |  |
|  | ≥40 |  | *20 (3.2)* |  |  |
| Civil status | In relationship |  | *650 (98.6)* |  |  |
| Nationality (born) | Faroe Islands |  | *573 (86.2)* |  |  |
|  | Other Nordic country |  | *67 (10.1)* |  |  |
|  | Europe |  | *6 (0.9)* |  |  |
|  | Outside Europe |  | *19 (2.9)* |  |  |
| Location | City |  | *299 (45.2)* |  |  |
|  | Village <6000 |  | *363 (54.8)* |  |  |
| BMI (pre-pregnancy) |  | 602 | 27.3 (5.3) | 26.4 | 15.4-50.9 |
| BMI groups | <18.5 |  | *3 (0.5)* |  |  |
|  | 18.5-24.9 |  | *217 (36.0)* |  |  |
|  | 25.0-29.9 |  | *234 (38.9)* |  |  |
|  | 30.0-34.9 |  | *91 (15.1)* |  |  |
|  | 35.0-39.9 |  | *43 (7.1)* |  |  |
|  | ≥40.0 |  | *14 (2.3)* |  |  |
| Education | Unskilled |  | *98(14.8)* |  |  |
|  | Low |  | *266 (40.2)* |  |  |
|  | Median |  | *193 (29.2)* |  |  |
|  | High |  | *105 (15.9)* |  |  |
| Smoke or snuff before pregnancy (Yes) | Yes |  | *168 (25.5)* |  |  |
| Smoke or snuff the past week | Yes |  | *58 (9.2)* |  |  |
| Smoke or snuff yesterday or today | Yes |  | *39 (6.2)* |  |  |
| Alcohol intake (during pregnancy) | Yes |  | *15 (2.5)* |  |  |
| Supplements yesterday with iodine included | Yes |  | *446 (81.2)* |  |  |
| Do you take iodine-containing supplements | Yes |  | *107 (17.4)* |  |  |
|  | No |  | *101 (16.4)* |  |  |
|  | Don’t know |  | *407 (66.2)* |  |  |
| Vegetarian | Total |  | *8 (1.2)* |  |  |
|  | Fish, milk or egg (yes) |  | *6 (0.9)* |  |  |
| Parity |  | 652 | 1.2 (1.1) | 1.0 | 1-8 |
| Gestational age |  | 646 | 21.4 (3.5) | 20 | 16-40 |
| Thyroid disease |  |  | *19 (3.3)* |  |  |
