# Supplementary material for: Iodine nutrition among pregnant women in the Faroe Islands
Source: Br J Nutr. 2024 Sep 16;132(4):495–502. doi: 10.1017/S0007114524001697 (PMC11499083; doi:10.1017/S0007114524001697)
Supplement: Johannesen et al. supplementary material 3 — Johannesen et al. supplementary material [file S0007114524001697sup003.docx]

| **Supplementary Table S2.** Reported dietary intake at enrolment for yesterday/today and the previous week. | | | | | | |
| --- | --- | --- | --- | --- | --- | --- |
| **Period** | **Food item** | ***n*** | **Mean (SD) or *n(%)*** | **95% CI** | **Median** | **Min-Max** |
| Yesterday/  today | Milk product intake (yes) |  | *490 (77.7)* |  |  |  |
|  | Milk products | 631 | 1.7 (1.2) | 1.6 ; 1.8 | 2.0 | 0-6 |
|  | Cheese intake (yes) |  | *314 (49.8)* |  |  |  |
|  | Cheeses | 631 | 0.6 (0.7) | 0.5 ; 0.6 | 0.0 | 0-3 |
|  | Fish dinner (yes) |  | *116 (18.4)* |  |  |  |
|  | Fish cold cut (yes) |  | *73 (11.6)* |  |  |  |
|  | Egg (yes) |  | *138 (21.9)* |  |  |  |
|  | Bread (yes) ^*^ |  | *253 (89.1)* |  |  |  |
| Past Week | Milk products^†^ | 629 | 19.9 (12.8) | 18.9 ; 21.0 | 17.5 | 0-71 |
|  | Cheese^‡^ | 630 | 5.0 (5.0) | 4.6 ; 5.4 | 5.0 | 0-56 |
|  | Fish dinner | 628 | 1.6 (1.5) | 1.5 ; 1.7 | 1.0 | 0.8 |
|  | Fish cold cut^‡^ | 625 | 2.0 (3.0) | 1.9 ; 2.3 | 0.0 | 0-28 |
|  | Eggs | 622 | 2.3 (2.7) | 2.1 ; 2.5 | 2.5 | 0-28 |
|  | Bread^*^*^,^* ^§^ | 284 | 7.6 (5.2) | 7.0 ; 8.2 | 7.0 | 0.17.5 |
| Iodised salt | Yes |  | *348 (55.8)* |  |  |  |
|  | No |  | *92 (14.7)* |  |  |  |
|  | Don’t know |  | *184 (29.5)* |  |  |  |
| Seaweed | Past year (yes) |  | *292 (48.6)* |  |  |  |
|  | Past week (yes) |  | *54 (8.7)* |  |  |  |
| Times during the past 12 months | Whale meat | 353 | 3.7 (5.4) | 3.1 ; 4.3 | 2.5 | 0-52 |
|  | Whale blubber | 224 | 3.3 (3.3) | 2.9 ; 3.8 | 2.5 | 0-29 |
|  | Seabirds | 389 | 2.5 (2.6) | 2.2 ; 2.8 | 0.2 | 0.24 |
| Times during pregnancy^\|^ | Whale meat | 315 | 0.7 (1.8) | 0.5 ; 0.9 | 0.0 | 0-15 |
|  | Whale blubber | 200 | 0.7 (1.8) | 0.5 ; 1.0 | 0.0 | 0-10 |
|  | Seabirds | 348 | 1.5 (2.9) | 1.2 ; 1.7 | 0.0 | 0-16 |
| ^*^Bread as a food item was included in the questionnaire 11 months after the project start-up. ^†^One outlier removed. ^‡^Counted as slices of bread with this food item on top. ^§^Slices of bread. ^\|^Until enrolment | | | | | | |
