# Supplementary material for: Iodine nutrition among pregnant women in the Faroe Islands
Source: Br J Nutr. 2024 Sep 16;132(4):495–502. doi: 10.1017/S0007114524001697 (PMC11499083; doi:10.1017/S0007114524001697)
Supplement: Johannesen et al. supplementary material 4 — Johannesen et al. supplementary material [file S0007114524001697sup004.docx]

| Supplementary Table S3. Univariable linear regression model: fractional change in UIC (µg/L) per unit change in the independent variable. | | | | | | | | | |
| --- | --- | --- | --- | --- | --- | --- | --- | --- | --- |
| **Variable** | **Group** | **n** | **Ratio**^**^ | **95% CI** | ***p*-value** | **Standardized-*β*** | **R^2^** | **F** | **P-value** |
| **Age** |  | 616 | 1.01 | 1.0;1.02 | 0.02 | 1.23 | 0.008 | 5.1 | **0.02** |
| Age group | <25 | 76 | - | - | - | - | 0.02 | 2.6 | **0.04** |
|  | 25-29 | 195 | 0.95 | 0.79;1.14 | 0.6 | 0.92 |  |  |  |
|  | 30-34 | 215 | 1.14 | 0.95;1.36 | 0.2 | 1.23 |  |  |  |
|  | 35-39 | 112 | 1.08 | 0.89;1.32 | 0.5 | 1.10 |  |  |  |
|  | ≥40 | 19 | 1.32 | 0.94;1.87 | 0.1 | 1.18 |  |  |  |
| **Education** | Up to upper secondary | 199 | - |  |  |  | 0.005 | 3.2 | 0.08 |
|  | Skilled and higher | 435 | 1.1 | 0.99;1.24 | 0.08 | 1.18 |  |  |  |
| Smoke or snuff^*^ |  | 56 | 0.87 | 0.72;1.05 | 0.2 | 0.88 | 0.003 | 2.0 | 0.2 |
| Alcohol intake^*^ |  | 15 | 0.84 | 0.59;1.19 | 0.3 | 0.91 | 0.002 | 0.9 | 0.3 |
| Place at birth | Faroe Islands | 550 | - | - | - | - | 0.0004 | 0.12 | 0.9 |
|  | Nordic country | 67 | 0.97 | 0.81;1.15 | 0.7 | 0.96 |  |  |  |
|  | Outside Nordic region | 24 | 1.04 | 0.79;1.37 | 0.8 | 1.03 |  |  |  |
| **Residence** | Capital area | 290 | - | - | - | - | 0.005 | 2.9 | **0.09** |
|  | Outside capital area (<6000) | 349 | 0.91 | 0.82;1.01 | 0.09 | 0.86 |  |  |  |
| BMI |  | 594 | 1.0 | 0.99;1.01 | 0.6 | 0.95 | 0.001 | 0.3 | 0.6 |
| BMI Group | <25,0 | 218 | - | - | - | - | 0.001 | 0.08 | 0.9 |
|  | 25,0-29,9 | 231 | 0.98 | 0.87;1.12 | 0.8 | 0.97 |  |  |  |
|  | 30,0-34,9 | 89 | 0.96 | 0.81;1.14 | 0.7 | 0.95 |  |  |  |
|  | 35,0-39,9 | 43 | 1.01 | 0.81;1.27 | 0.9 | 1.01 |  |  |  |
|  | ≥40,0 | 14 | 0.95 | 0.65;1.37 | 0.8 | 0.97 |  |  |  |
| **Vitamins**^†^*^,^* ^‡^ |  | 431 | 1.40 | 1.22;1.61 | <0.001 | 1.58 | 0.04 | 22.4 | **<0.001** |
| Milk products^†^ |  | 482 | 1.10 | 0.97;1.25 | 0.13 | 0.15 | 0.004 | 2.1 | 0.1 |
| **Cheese**^†^ |  | 311 | 1.18 | 1.06;1.31 | 0.003 | 1.32 | 0.02 | 9.2 | **0.003** |
| **Fish dinner**^†^ |  | 113 | 1.43 | 1.24;1.63 | <0.001 | 1.58 | 0.04 | 25.9 | **<0.001** |
| **Fish cold-cut**^†^ |  | 73 | 1.26 | 1.07;1.49 | 0.006 | 1.29 | 0.01 | 7.7 | **0.006** |
| **Egg**^†^ |  | 136 | 1.14 | 1.005;1.30 | 0.04 | 1.21 | 0.007 | 4.1 | **0.04** |
| Bread^†^*^,^* ^§^ |  | 251 | 1.04 | 0.82;1.32 | 0.8 | 1.04 | 0.0004 | 0.10 | 0.8 |
| Milk products^\|^ |  | 621 | 1.002 | 1.0;1.005 | 0.4 | 1.08 | 0.001 | 0.8 | 0.4 |
| Cheese^\|^ |  | 621 | 1.0006 | 1.0;1.01 | 0.9 | 1.01 | 0.00 | 0.03 | 0.9 |
| **Fish dinner**^\|^ |  | 619 | 1.04 | 1.005;1.08 | 0.03 | 1.23 | 0.008 | 4.9 | **0.03** |
| Fish cold-cut^\|^ |  | 616 | 1.01 | 1.0;1.03 | 0.1 | 1.16 | 0.004 | 2.6 | 0.1 |
| **Egg**^\|^ |  | 613 | 1.02 | 1.0;1.04 | 0.06 | 1.19 | 0.006 | 3.7 | **0.06** |
| Bread^§^*^,^* ^\|^ |  | 280 | 1.0 | 1.0;1.005 | 0.5 | 0.92 | 0.001 | 0.4 | 0.5 |
| Seaweed^¶^ | During past week | 53 | 1.03 | 0.85;1.25 | 0.8 | 1.03 | 0.0001 | 0.08 | 0.8 |
| Whale meat | Times previous 12 months | 346 | 1.002 | 1.0;1.02 | 0.6 | 1.06 | 0.001 | 0.2 | 0.6 |
| Whale meat | Since fecundity | 308 | 0.95 | 0.95;1.03 | 0.5 | 0.92 | 0.001 | 0.4 | 0.5 |
| Whale blubber | Times previous 12 months | 217 | 1.007 | 0.98;1.04 | 0.7 | 1.07 | 0.001 | 0.2 | 0.7 |
| Whale blubber | Since fecundity | 195 | 1.0 | 0.94;1.05 | 0.9 | 0.97 | 0.001 | 0.02 | 0.9 |
| Seabirds | Times previous 12 months | 374 | 0.98 | 0.95;1.007 | 0.1 | 0.84 | 0.006 | 2.2 | 0.1 |
| Seabirds | Since fecundity | 338 | 0.98 | 0.96;1.01 | 0.2 | 0.86 | 0.004 | 1.4 | 0.2 |
| **PC-1** | Fish, cold-cut, egg | 609 | 1.08 | 1.02;1.14 | 0.006 | 1.29 | 0.01 | 7.5 | **0.006** |
| PC-2 | Milk products and cheese | 609 | 1.009 | 0.96;1.07 | 0.7 | 1.04 | 0.0002 | 0.13 | 0.7 |
| ^*^During pregnancy and yesterday/today (yes).  ^†^Intake yesterday/today (yes).  ^‡^Multivitamines containing iodine.  ^§^Bread as a food item was included in the questionnaire 11 months after the project started  ^\|^Intake during the previous week (#times).  ^¶^Intake during the past week “Yes”.  ^**^Corresponds to 10*^B^*, with *B* the unstandardized regression coefficient | | | | | | | | | |
