# Supplementary material for: Iodine nutrition among pregnant women in the Faroe Islands
Source: Br J Nutr. 2024 Sep 16;132(4):495–502. doi: 10.1017/S0007114524001697 (PMC11499083; doi:10.1017/S0007114524001697)
Supplement: Johannesen et al. supplementary material 5 — Johannesen et al. supplementary material [file S0007114524001697sup005.docx]

| Supplementary Table S4. PCA scores of five food groups recorded in the 2^nd^ trimester (mean gestational age 20.5 weeks) representing the previous week’s intake^*^*^.^*^†^. | | |
| --- | --- | --- |
| **Food groups** | **PC-1 (30.7%)** | **PC-2 (20.1%)** |
| Fish cold-cut | 0.719 | 0.213 |
| Fish dinners | 0.674 | 0.106 |
| Egg | 0.590 | -0.109 |
| Cheese | -0.115 | 0.809 |
| Dairy products | -0.255 | 0.649 |
| ^*^The PCA is based on Eigenvalues >1 and Varimax rotation.  ^†^Highlighted scores (also called) loadings indicate that the variance in the corresponding element item contributes substantially to the variance summarised by the principal component (PC). | | |
