# Supplementary material for: Iodine nutrition among pregnant women in the Faroe Islands
Source: Br J Nutr. 2024 Sep 16;132(4):495–502. doi: 10.1017/S0007114524001697 (PMC11499083; doi:10.1017/S0007114524001697)
Supplement: Johannesen et al. supplementary material 6 — Johannesen et al. supplementary material [file S0007114524001697sup006.docx]

| **Supplementary Table S5**  Differences in Urinary Iodine Concentration with time, and predicted personal and dietary values. | | | | | | | | |  |
| --- | --- | --- | --- | --- | --- | --- | --- | --- | --- |
|  | **June 2020 – November 2020** | | **December 2020 – May 2021** | | **June 2021 – November 2021** | | **December 2021 – April 2022** | |  |
|  | **n=177 (28.1%)** | | **n=179 (28.5%)** | | **n=158 (25.1%)** | | **n=115 (18.3%)** | |  |
| Variable | **Median (IQR), Mean (SD) or *n(%)*** | **95% CI** | **Median (IQR), Mean (SD) or *n(%)*** | **95% CI** | **Median (IQR), Mean (SD) or *n(%)*** | **95% CI** | **Median (IQR), Mean (SD) or *n(%)*** | **95% CI** | **p-value** |
| Iodine^*^ | 117 (84-183) | 106-129 | 107 (73-183) | 100-127 | 100 (71-150) | 91-114 | 101 (68-143) | 88-116 | 0.01^§^ |
| Age^†^ | 30.3 (4.7) | 29.6-31.0 | 30.7 (4.7) | 30.0-31.4 | 30.3 (5.3) | 29.5-31.1 | 30.0 (5.2) | 29.0-30.9 | 0.7^\|^ |
| Gest week^*^ | 20.5 (2.2) | 16.0-34.0 | 20.0 (1.7) ^\|\|^ | 16.0-35.0 | 20.7 (1.9) | 17.0-28.0 | 20.8 (1.9) | 17.0-30.0 | <0.001^\|^ |
| BMI^*^ | 26.2 (23.6-30.9) | 25.2-26.9 | 26.9 (23.8-29.8) | 26.1-27.9 | 26.6 (24.3-30.0) | 25.7-27.4 | 25.4 (23.1-28.7) | 24.5-26.8 | 0.2 |
| Fish total last week (#times) ^*^ | 3.0 (1.0-5.5) | 2.5-3.5 | 3.0 (1.0-5.5) | 2.0-3.5 | 2.5 (1.0-5.5) | 2.0-3.5 | 2.0 (1.0-4.5) | 1.0-3.0 | 0.2^§^ |
| Dairy products last week (# times)*^1^* | 24.5 (15-33) | 21-27 | 24.5 (15-35) | 21-28 | 21.5 (13-34) | 17-25 | 22.5 (15-33) | 20-25 | 0.7^§^ |
| Median total food intake last week (#times)^*^ | 31 (20-39) | 27-35 | 31 (21-41) | 28-33 | 33 (22-43) | 28-36 | 33 (24-46) | 31-37 | 0.1^§^ |
| Median food recording yesterday (#times “yes”) ^*^ | 3 (2-4) | 3-3 | 2 (1-4) | 2-3 | 4 (2-5) | 3-4 | 4 (3-5) | 3-4 | <0.001^§^ |
| Fish products yesterday (yes) ^‡^ | 54 (30.5) |  | 42 (23.5) |  | 41 (25.9) |  | 26 (22.6) |  | 0.4^¶^ |
| Dairy products yesterday (yes) ^‡^ | 152 (85.9) |  | 132 (73.7) |  | 134 (84.8) |  | 98 (85.2) |  | 0.008^¶^ |
| Vitamin with iodine (yes) ^‡^ | 128 (83.7) |  | 119 (81.5) |  | 120 (81.6) |  | 79 (76.7) |  | 0.6^¶^ |
| ^*^Median values (IQR). ^†^Mean values (SD). ^‡^number and percent. ^§^p-value based on the Kruskal-Wallis test. ^\|^p-value based on ANOVA test (^\|\|^only group two (Dec. 202-May 21) was significantly different from the other three groups, analysis based on the Bonferroni PostHoc test. ^¶^Based on Pearson Chi-square test. | | | | | | | | | |
